# Improving Junior Doctor Confidence in Physical Health Examination in Mental Health Inpatient Settings: A Quality Improvement Project

**DOI:** 10.1192/bjo.2026.11475

**Published:** 2026-06-30

**Authors:** Sara Salama, Ekaterina Duokova

**Affiliations:** 1 Lancashire and South Cumbria Foundation Trust, Preston, United Kingdom; 2 North London Foundation Trust, London, United Kingdom

## Abstract

**Aims:**

This quality improvement project aimed to improve junior doctors’ confidence and consistency in performing physical health examinations within a mental health hospital. Additional objectives included improving awareness of escalation pathways to acute medical services and supporting safer, more timely identification of physical health concerns.

**Methods:**

The project was conducted at Chase Farm Hospital, part of North London NHS Foundation Trust, which includes older adult, general adult, and forensic inpatient wards. A baseline questionnaire was distributed to junior doctors (Foundation Year, GP trainees, and Core Psychiatry trainees) to assess prior training, confidence levels, and awareness of escalation thresholds when managing physical health issues in psychiatric settings. Based on the findings, a Plan–Do–Study–Act (PDSA) cycle was implemented. Two structured flowcharts were developed: one outlining the expected components of physical examinationin a mental health setting, and another detailing criteria and processes for referral to Accident & Emergency. These resources were disseminated electronically, and a follow-up questionnaire was used to evaluate their perceived usefulness and relevance.

**Results:**

Eleven doctors completed the baseline questionnaire. Eight reported low confidence in performing physical examinations in mental health settings, and all respondents felt that additional structured teaching at induction would be beneficial. Six doctors completed the follow-up questionnaire after introduction of the flowcharts. All respondents found the flowcharts helpful and agreed they would have been valuable at the start of their rotation. There was unanimous support for including the resources in junior doctor induction and displaying them in on-call areas.

**Conclusion:**

This quality improvement project demonstrates that brief, targeted educational tools can significantly improve junior doctor confidence and clarity regarding physical health assessment and escalation in psychiatric inpatient settings. Embedding structured guidance into induction programmes has the potential to enhance patient safety, trainee experience, and multidisciplinary collaboration. Future work will focus on Trust-wide implementation and evaluation of longer-term clinical impact.

